# Improvement in clinical features of hypercortisolism during osilodrostat treatment: findings from the Phase III LINC 3 trial in Cushing's disease

**DOI:** 10.1007/s40618-024-02359-6

**Published:** 2024-05-02

**Authors:** R. Pivonello, M. Fleseriu, J. Newell-Price, A. Shimatsu, R. A. Feelders, P. Kadioglu, A. Tabarin, T. C. Brue, E. B. Geer, A. Piacentini, A. M. Pedroncelli, B. M. K. Biller

**Affiliations:** 1 Dipartimento di Medicina Clinica e Chirurgia, Sezione di Endocrinologia, Diabetologia, Andrologia e Nutrizione, Università Federico II di Napoli, Naples, Italy; 2 Pituitary Center, Departments of Medicine and Neurological Surgery, Oregon Health & Science University, Portland, OR USA; 3 Department of Oncology and Metabolism, The Medical School, University of Sheffield, Sheffield, UK; 4 Advanced Medical Care Center, Omi Medical Center, Kusatsu, Japan; 5 Department of Internal Medicine, Endocrine Section, Erasmus Medical Center, Rotterdam, Netherlands; 6 Division of Endocrinology, Metabolism and Diabetes, Cerrahpasa Medical Faculty, Istanbul University-Cerrahpasa, Istanbul, Turkey; 7 CHU de Bordeaux, Bordeaux, France; 8 Aix-Marseille Université, Institut National de la Santé et de la Recherche Médicale, Marseille Medical Genetics, and Assistance Publique Hôpitaux de Marseille, Department of Endocrinology, Hôpital de la Conception, Centre de Référence des Maladies Rares de l’Hypophyse, Marseille, France; 9 Multidisciplinary Pituitary & Skull Base Tumor Center, Memorial Sloan Kettering Cancer Center, New York, NY USA; 10 Recordati SpA, Milan, Italy; 11 Recordati AG, Basel, Switzerland; 12 Camurus AB, Lund, Sweden; 13 Neuroendocrine and Pituitary Tumor Clinical Center, Massachusetts General Hospital, Boston, MA USA

**Keywords:** Cushing’s disease, Diabetes, Dyslipidaemia, Hypertension, Osilodrostat, Urinary free cortisol

## Abstract

**Purpose:**

Cushing’s disease is associated with substantial morbidity and impaired quality of life (QoL) resulting from excess cortisol exposure. The current study explored improvements in clinical signs and additional specific manifestations of hypercortisolism during osilodrostat (potent oral 11β-hydroxylase inhibitor) therapy by degree of control of mean urinary free cortisol (mUFC).

**Methods:**

LINC 3 (NCT02180217) was a prospective, open-label, 48-week study of osilodrostat (starting dose: 2 mg bid; maximum: 30 mg bid) that enrolled 137 adults with Cushing’s disease and mUFC > 1.5 times the upper limit of normal (ULN). mUFC (normal range 11‒138 nmol/24 h), cardiometabolic parameters (blood pressure, weight, waist circumference, body mass index, total cholesterol, fasting plasma glucose, glycated haemoglobin), physical manifestations of hypercortisolism (facial rubor, striae, fat distribution, bruising, hirsutism [females], muscle atrophy) and QoL were evaluated. mUFC was defined as controlled if ≤ ULN, partially controlled if > ULN but ≥ 50% reduction from baseline, and uncontrolled if > ULN and < 50% reduction from baseline. Concomitant medications were permitted throughout the study.

**Results:**

At weeks 24 and 48, respectively, mUFC was controlled in 93 (67.9%) and 91 (66.4%) patients, partially controlled in 20 (14.6%) and 13 (9.5%), and uncontrolled in 24 (17.5%) and 33 (24.1%). Overall, mean improvements from baseline in cardiometabolic at week 24 were greater in patients with controlled or partially controlled versus uncontrolled mUFC; at week 48, improvements occurred irrespective of mUFC control. Generally, physical manifestations and QoL progressively improved from baseline irrespective of mUFC control.

**Conclusions:**

Improvements in clinical signs and additional specific manifestations of hypercortisolism associated with Cushing’s disease occurred alongside decreases in mUFC.

*Trial registration* NCT02180217 (first posted July 2014).

**Supplementary Information:**

The online version contains supplementary material available at 10.1007/s40618-024-02359-6.

## Introduction

Cushing’s disease, the most common form of endogenous Cushing’s syndrome, is a rare and debilitating condition caused by an adrenocorticotropic hormone (ACTH)-secreting pituitary tumour, resulting in excessive secretion of cortisol from the adrenal glands [1]. The subsequent exposure to elevated cortisol levels is associated with a wide range of comorbidities, impaired health-related quality of life (HRQoL) and, especially in the case of untreated disease, increased mortality [2]. Many clinical features and physical manifestations are associated with Cushing’s disease. Clinical complications include visceral obesity, hypertension, impaired glucose metabolism (including diabetes) and dyslipidaemia [3, 4]; these complications result in an increased risk of cardiovascular disease, one of the main causes of death in patients with Cushing’s disease [2, 5]. Dermatol-ogical manifestations of hypercortisolism, most commonly including skin thinning with purple striae, ecchymoses and hirsutism (in females), together with fatigue and proximal myopathy, are also commonly associated with Cushing’s disease [2, 3]. Multiple comorbidities are a defining feature of patients with Cushing’s disease [6] and contribute to impaired patient HRQoL [7], which may persist even following remission [8]. Osilodrostat is an oral adrenal steroidogenesis inhibitor that targets 11β-hydroxylase (the enzyme that catalyses the final step of cortisol synthesis) and is currently approved for the treatment of patients with Cushing’s disease (USA) or endogenous Cushing’s syndrome (EU and Japan) who are eligible for medical therapy [9–11].

The current report explores changes in clinical signs and additional specific manifestations of hypercortisolism in patients with Cushing’s disease following medical treatment with osilodrostat. Osilodrostat treatment led to rapid and sustained normalisation of mean urinary free cortisol (mUFC) in many patients during the 48 week core phase of the Phase III LINC 3 study [12], which continued into the long-term extension [13]. The majority of enrolled patients (n = 132/137, 96.4%) achieved normal mUFC (below the upper limit of normal [ULN]) at least once during the study, and more patients maintained a complete response with osilodrostat versus with placebo following the 8 week randomised-withdrawal period (n = 31 [86.1%] vs n = 10 [28.6%]; odds ratio 13.7 [95% confidence interval (CI) 3.7–53.4]; *P* < 0.0001) [12]. Late-night salivary cortisol (LNSC) was also measured; mean levels rapidly decreased within the first 12 weeks, then remained below baseline values [12]. However, LNSC data were not recorded for all patients at all time points, precluding meaningful analyses of outcomes in patients with control of mUFC and/or LNSC in the LINC 3 study alone. mUFC is regularly used to evaluate the efficacy of medical therapies in patients with Cushing’s disease, and achieving mUFC normalisation is a key treatment goal [5]. Furthermore, if mUFC improves but does not normalise, alternative medical therapy options may be considered [5]. Changes in clinical features of hypercortisolism according to the degree of mUFC control achieved by each patient are examined in this report.

## Methods

### Patients and study design

Full details of the study design for the 48 week core phase of the LINC 3 study have been described previously [12]. Briefly, adult patients with confirmed Cushing’s disease and mUFC (calculated from three 24 h urine samples) > 1.5 × ULN were enrolled. All patients received osilodrostat (starting dose: 2 mg twice daily [bid]; maximum dose: 30 mg bid) throughout the study, except those randomised to placebo during an 8-week randomised-withdrawal phase after receiving osilodrostat for 26 weeks (weeks 26–34). Dose titration was permitted based on efficacy (to achieve the goal of mUFC ≤ ULN) and tolerability. Concomitant medications were permitted throughout the study, including medications for the treatment of hypertension, diabetes and dyslipidaemia. Patients were classified as having hypertension if they had one or more of the following: history of antihypertensive medication; medical history of hypertension; baseline systolic blood pressure (SBP) > 130 mmHg; baseline diastolic blood pressure (DBP) > 90 mmHg. Glycaemic status was defined as follows based on fasting plasma glucose (FPG): normoglycaemia, FPG < 100 mg/dL; impaired fasting glucose, FPG 100– < 126 mg/dL; diabetic, FPG ≥ 126 mg/dL. Patients could also be classified as having diabetes at baseline if they had one or more of the following: history of antidiabetic medication; medical history of diabetes mellitus; glycated haemoglobin (HbA_1c_) ≥ 6.5%; FPG ≥ 126 mg/dL. Patients were classified as having dyslipidaemia if they had one or more of the following: history of lipid-lowering medication; medical history of dyslipidaemia; baseline total cholesterol ≥ 200 mg/dL (≥ 5.2 mmol/L); triglycerides > 150 mg/dL (> 1.7 mmol/L); high-density lipoprotein cholesterol (HDL-c) < 40 mg/dL (< 1.0 mmol/L) in male patients or < 50 mg/dL (< 1.3 mmol/L) in female patients; low-density lipoprotein cholesterol (LDL-c) > 100 mg/dL (> 2.6 mmol/L). The study was conducted in accordance with the Declaration of Helsinki, with an independent ethics committee/institutional review board at each site approving the study protocol. Patients provided written informed consent to participate. The LINC 3 study is registered at ClinicalTrials.gov (NCT02180217).

### Study objectives and assessments

In the present post hoc analysis from the LINC 3 study, changes from baseline in clinical features of hypercortisolism and HRQoL were assessed by degree of mUFC control. Mean 24 h UFC concentration (three 24 h urine samples) was determined using liquid chromatography-tandem mass spectrometry (normal range: 11–138 nmol/24 h [4–50 μg/24 h]) at baseline, then every 2–4 weeks (depending on the study period), as used in the assessment of the primary and secondary endpoints of the study [12]. Degree of mUFC control was defined as controlled (mUFC ≤ ULN), partially controlled (> ULN but ≥ 50% reduction from baseline) or uncontrolled (mUFC > ULN and < 50% reduction from baseline).

Physical examination, including measurement of body weight (with calculation of body mass index [BMI]), waist circumference, SBP and DBP, was conducted at baseline, then every 2–4 weeks (depending on the study period). SBP and DBP were recorded as the mean of three values at 1–2 min intervals after the patient had been sitting for 5 min (with back supported and both feet placed on the floor).

Physical features of hypercortisolism (facial rubor, hirsutism [in females], striae, bruising, proximal muscle wasting, central obesity, supraclavicular and dorsal fat pads) were assessed at baseline, then at the end of each study period (weeks 12, 24, 34 and 48). Two photographs were taken, one frontal and one lateral, from the shoulders up to assess facial rubor and supraclavicular and dorsal fat pads. Two additional photographs were taken, one frontal and one dorsal, of the trunk with the patient in a standing position to assess hirsutism, striae, proximal muscle atrophy, central obesity and bruising. Photographs were rated subjectively by local investigators (0 = absent; 1 = mild; 2 = moderate; 3 = severe).

Assessment of FPG, HbA_1c_ and cholesterol was performed at a central laboratory. FPG and HbA_1c_ were measured every 12 weeks, except during the randomised-withdrawal period, when measurements were taken every 2 weeks. Cholesterol was measured every 4 weeks throughout the study.

Bone mineral density (BMD; L1–L4 lumbar spine and total hip) was assessed by dual-energy X-ray absorptiometry and standardised against BMD T-score at baseline and week 48. Each patient was scanned with the same instrument throughout the study, and images were reviewed centrally.

HRQoL was measured using the CushingQoL questionnaire (scored from 12 [worst] to 60 [best]) [14] and the Beck Depression Inventory II (BDI-II; scored from 0 [best] to 63 [worst]) [15] prior to any clinical assessments, drug dosing or diagnostic testing at baseline, then at weeks 4, 8, 12, 24, 26, (28 for randomised patients only,) 30, (32 for randomised patients only,) 34 and 48.

### Statistical methods

All data were analysed descriptively. Mean change from baseline and corresponding 95% CIs were provided for clinical signs of hypercortisolism by degree of mUFC control at weeks 24 and 48. Correlations were evaluated using the Pearson’s correlation coefficient.

## Results

### Patient characteristics

Overall, 77.4% of patients enrolled in the study were female. Mean baseline mUFC was 7.3 × ULN (Table 1). All patients had at least one relevant medical history/current medical condition, which included hypertension, diabetes and dyslipidaemia (Table 1). At baseline, the most frequently reported physical manifestation rated as severe was central obesity (16.1% mild, 29.9% moderate, 25.5% severe). Additional physical manifestations were rated mostly as mild or moderate: supraclavicular fat pad (24.8% mild, 35.0% moderate, 8.8% severe); dorsal fat pad (32.1% mild, 34.3% moderate, 7.3% severe); facial rubor (34.3% mild, 23.4% moderate, 5.8% severe); hirsutism (females; 37.7% mild, 17.0% moderate, 3.8% severe); proximal muscle atrophy (30.7% mild, 15.3% moderate, 5.8% severe); striae (24.1% mild, 18.2% moderate, 6.6% severe); ecchymoses (22.6% mild, 11.7% moderate, 4.4% severe).

**Table 1:** Baseline clinical characteristics

	All patients (N = 137)
Median age, years (IQR)	40.0 (31.0‒49.0)
Female, n (%)	106 (77.4)
Mean mUFC, nmol/24 h(SD)	1006 (1590; 7.3 × ULN)
Median mUFC, nmol/24 h (range)	476 (314–919; 3.4 × ULN)
Mean weight, kg (SD)	80.8 (22.4)
Mean BMI, kg/m^2^ (SD)	30.3 (7.8)
Patients with diabetes,^a^ n (%)	61 (44.5)
Patients with hypertension,^b^ n (%)	119 (86.9)
Patients with dyslipidaemia,^c^ n (%)	115 (83.9)

*IQR* interquartile range, *SD* standard deviation

^a^History of antidiabetic medication, medical history of diabetes mellitus, HbA_1c_ ≥ 6.5%, or FPG ≥ 126 mg/dL

^b^History of antihypertensive medication, medical history of hypertension, baseline SBP > 130 mmHg, or baseline DBP > 90 mmHg

^c^History of lipid-lowering medication, medical history of dyslipid-aemia, baseline total cholesterol ≥ 200 mg/dL (≥ 5.2 mmol/L), triglycerides > 150 mg/dL (> 1.7 mmol/L), HDL-c < 40 mg/dL (< 1.0 mmol/L) in male patients or < 50 mg/dL (< 1.3 mmol/L) in female patients, or LDL-c > 100 mg/dL (> 2.6 mmol/L)

### Effect of osilodrostat on mUFC

**Fig. 1:**
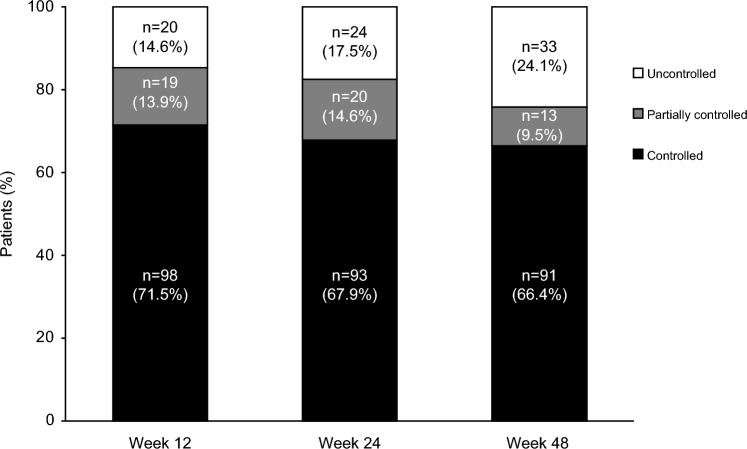
Proportion of patients with controlled, partially controlled and uncontrolled mUFC over time. Based on patients in the full analysis set (N = 137). Patients who had discontinued or otherwise had missing mUFC values at a given visit were counted as uncontrolled. Controlled, mUFC ≤ ULN; partially controlled, mUFC > ULN but ≥ 50% reduction from baseline; uncontrolled, mUFC > ULN and < 50% reduction from baseline

**Fig. 2:**
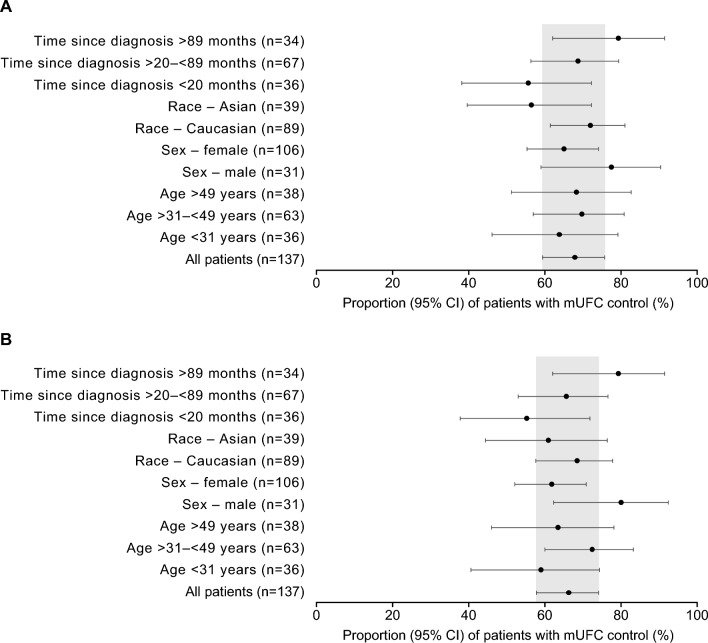
Overview of mUFC control by age, sex, race and time since diagnosis at A) 24 and B) 48 weeks. mUFC control defined as mUFC ≤ ULN; shaded area indicates the ‘all patients’ data

Median duration of osilodrostat exposure from baseline to core study data cut-off was 75 weeks (range 1–165, IQR 48–117), and average median daily osilodrostat dose was 7.1 mg/day (range 1.1–53.9, IQR 3.8–14.0). The majority (71.5%) of patients achieved control of mUFC (≤ ULN) by week 12, with a further 13.9% achieving partial control. The proportion of patients with mUFC ≤ ULN remained high throughout the core study (Fig. 1). Overall, 132 (96.4%) patients achieved control of mUFC at least once during the 48 weeks. Descriptive analyses of the proportion of patients with mUFC control by age, sex, race and time since diagnosis suggest that these factors do not affect response to treatment at weeks 24 and 48 (Fig. 2; Supplementary Table 1).

### Changes in blood pressure

**Fig. 3:**
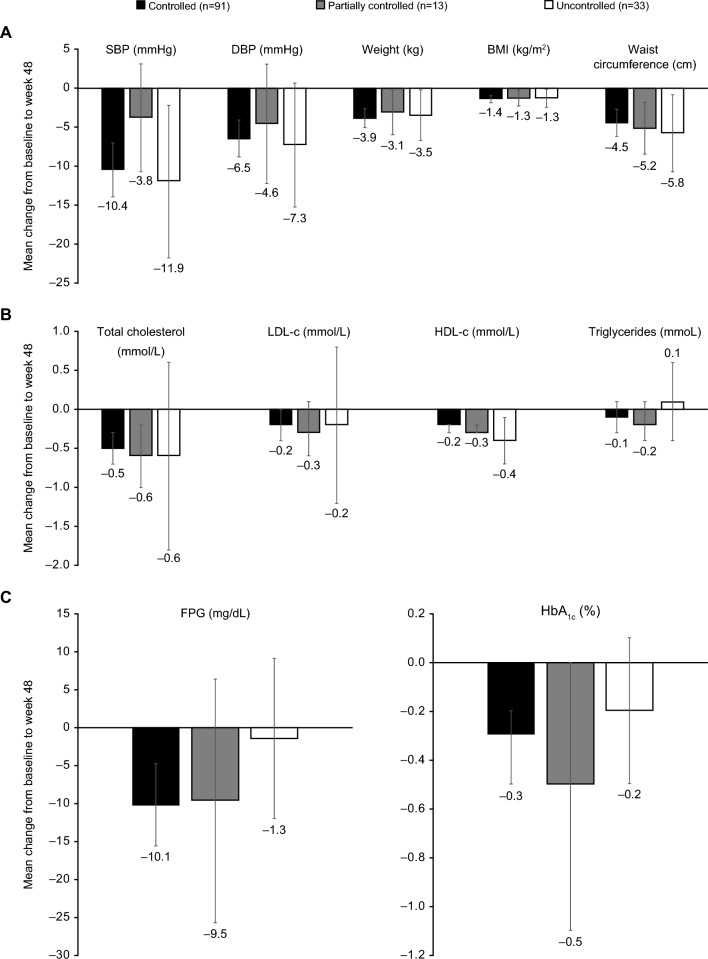
Mean (95% CI) change from baseline to week 48 in A) clinical signs, B) lipid profile, and C) glycaemic parameters by degree of mUFC control at week 48

In total, 86.9% of patients were classified as hypertensive at baseline (Table 1). Of patients with baseline SBP > 130 mmHg (n = 79), 58.2, 50.6 and 49.4% had SBP ≤ 130 mmHg after 12, 24 and 48 weeks of osilodrostat treatment, respectively; of patients with baseline DBP > 90 mmHg (n = 50), 72.0, 62.0 and 66.0% had DBP ≤ 90 mmHg at weeks 12, 24 and 48, respectively. In patients without hypertension at baseline, both SBP and DBP generally remained stable during osilodrostat treatment. At week 48, five patients with baseline SBP ≤ 130 mmHg had SBP > 130 mmHg, and four patients with baseline DBP ≤ 90 mmHg had DBP > 90 mmHg. Of all patients classified as hypertensive at baseline, at week 48, mean (95% CI) SBP and DBP improved both in patients who did and in those who did not receive antihypertensive medications during the study (SBP − 8.7 [− 12.6, − 4.8] and − 14.5 [− 20.1, − 9.0] mmHg, respectively; DBP − 5.1 [− 7.7, − 2.5] and − 10.2 [− 14.5, − 6.0] mmHg, respectively). Overall, at week 48, 40% (n = 34/85) of patients taking antihypertensive medication at baseline had either stopped or reduced the dose; 40% (n = 34/85) had an increase in dose of antihypertensive medication or number of medications. Of patients with controlled, partially controlled and uncontrolled mUFC at week 48, 43.1% (n = 25/58), 33.3% (n = 3/9) and 33.3% (n = 6/18), respectively, had either stopped or reduced the dose of antihypertensive medication; 39.7% (n = 23/58), 44.5% (n = 4/9) and 38.9% (n = 7/18), respectively, had an increase in dose or number of medications, and 17.2% (n = 10/58), 22.2% (n = 2/9) and 27.8% (n = 5/18), respectively, had no change. Clinically relevant improvements from baseline in mean (95% CI) SBP and DBP were noted by week 24 in patients with controlled (− 7.1 [− 10.3, − 3.9] and − 4.9 [− 7.1, − 2.7] mmHg, respectively) and partially controlled mUFC (− 7.2 [− 14.0, − 0.4] and − 5.2 [− 9.8, − 0.6] mmHg), but not in patients with uncontrolled mUFC (2.0 [− 9.7, 13.7] and 5.3 [− 3.3, 13.9] mmHg). By week 48, improvements in both SBP and DBP were seen irrespective of mUFC control (Fig. 3A). There was no correlation between change from baseline in mUFC and change from baseline in SBP or DBP at week 24 (r = − 0.02, *P* = 0.8326 and r = 0.02, *P* = 0.8160, respectively) and a weak correlation at week 48 (r = 0.20, *P* = 0.0433 and r = 0.18, *P* = 0.0715, respectively).

### Changes in body weight, waist circumference and BMI

Most patients had physical evidence of central obesity at baseline; mean weight was 80.8 kg and mean BMI was 30.3 kg/m^2^ (Table 1). Clinically relevant improvements in mean (95% CI) weight, waist circumference and BMI were noted by week 24 in patients with controlled mUFC (− 2.7 [− 3.6, − 1.8] kg, − 3.4 [− 4.9, − 1.8] cm and − 1.1 [− 1.4, − 0.7] kg/m^2^, respectively); improvements were not clinically relevant in patients with partially controlled mUFC (− 1.7 [− 3.8, 0.3] kg, − 2.4 [− 5.0, 0.3] cm and − 0.6 [− 1.4, 0.2] kg/m^2^) or uncontrolled mUFC (− 1.6 [− 3.5, 0.3] kg, 0.1 [− 2.4, 2.6] cm and − 0.6 [− 1.4, 0.1] kg/m^2^). By week 48, improvements from baseline were seen irrespective of mUFC control (Fig. 3A). No correlation was observed between change in mUFC and change in mean body weight, waist circumference or BMI at either week 24 or week 48 (Supplementary Table 2).

### Changes in lipid profile

In total, 83.9% of patients had dyslipidaemia at baseline (Table 1). Mean changes in cholesterol (including LDL-c and HDL-c) and triglycerides were similar across mUFC response subgroups at both week 24 and week 48 (Fig. 3B). There were no strong correlations between change in mUFC and change in total cholesterol, LDL-c, HDL-c or triglycerides at either week 24 or week 48 (Supplementary Table 2).

### Changes in glycaemic status

At baseline, 61 (44.5%) patients were classified as diabetic. Based on FPG levels only, most (67.9%) patients had normoglycaemia (FPG < 100 mg/dL) at baseline; 26.3% of patients had either impaired fasting glucose or diabetes (FPG ≥ 100 mg/dL). Of patients with baseline FPG ≥ 100 mg/dL (n = 36), 58.3, 63.8 and 44.4% had FPG < 100 mg/dL by weeks 12, 24 and 48, respectively. In total, at week 48, 48.8% (n = 21/43) of patients taking antidiabetic medication at baseline had stopped or reduced the dose, and 23.3% (n = 10/43) increased the dose or number of medications. Of patients with controlled, partially controlled and uncontrolled mUFC at week 48, 55.6% (n = 15/27), 75% (n = 3/4) and 25% (n = 3/12), respectively, had either stopped or reduced the dose of antidiabetic medication; 18.5% (n = 5/27), 25% (n = 1/4) and 33.3% (n = 4/12), respectively, had an increase in dose or number of medications, and 25.9% (n = 7/27), 0% (n = 0/4) and 41.7% (n = 5/12), respectively, had no change. Improvements in mean (95% CI) FPG and HbA_1c_ at week 24 occurred irrespective of mUFC control, although they were more evident in patients with controlled mUFC (− 13.3 [− 18.9, − 7.7] mg/dL and − 0.3% [− 0.5, − 0.2], respectively) or partially controlled mUFC (− 21.2 [− 37.0, − 5.4] mg/dL and − 0.4% [− 0.9, 0.0]) than in those with uncontrolled mUFC (− 7.4 [− 17.1, 2.4] mg/dL and − 0.0% [− 0.3, 0.3]). A similar pattern of mean changes in FPG and HbA_1c_ was observed at week 48 (Fig. 3C). There was a weak correlation between change from baseline in mUFC and change from baseline in FPG (r = 0.25, *P* = 0.008) and HbA_1c_ (r = 0.23, *P* = 0.012) at week 24. At week 48, the correlation was stronger for FPG (r = 0.33, *P* = 0.001) but absent for HbA_1c_ (r = 0.14, *P* = 0.161).

### Changes in BMD

Mean baseline L1‒L4 lumbar spine and total hip BMD in all patients was 1.0 and 0.9 g/cm^2^, respectively. Mean change from baseline to week 48 in BMD at both the lumbar spine and total hip was 0.0 g/cm^2^ in both male and female patients; as a mean percentage change from baseline (95% CI), the increase in BMD was more pronounced in males (lumbar spine: + 3.9% [− 0.2, 8.0]; total hip: + 1.7% [− 0.5, 4.0]) than females (lumbar spine: + 2.7% [1.3, 4.1]; total hip: − 0.1% [− 1.6, 1.3]). The degree of mUFC response had no effect on outcomes (Supplementary Table 3).

### Changes in physical manifestations of hypercortisolaemia

**Fig. 4:**
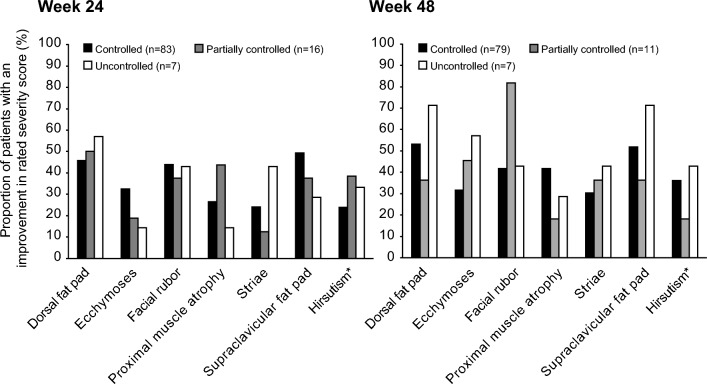
Proportion of patients with improvements in rated severity score from baseline for physical manifestations of hypercortisolism by degree of mUFC control at week 24 and week 48. An improvement was defined as the symptom score being lower (ie less severe) than at baseline. The denominator for the percentage is the number of patients in the full analysis set (all enrolled patients who received at least one dose of osilodrostat) with data available at both baseline and the given visit. *Females only

Physical manifestations of hypercortisolaemia were prevalent at baseline. At weeks 24 and 48, improvements in rated severity score from baseline occurred across all physical manifestations, with few patients rated with worsening scores. Improvements in physical manifestation severity scores were seen irrespective of the degree of mUFC response, with the exception of proximal muscle atrophy at week 24, whereby a numerically larger proportion of patients with controlled (26.5%) and partially controlled (43.8%) mUFC had improved severity scores compared with uncontrolled patients (14.3%; Fig. 4).

### Changes in HRQoL

**Fig. 5:**
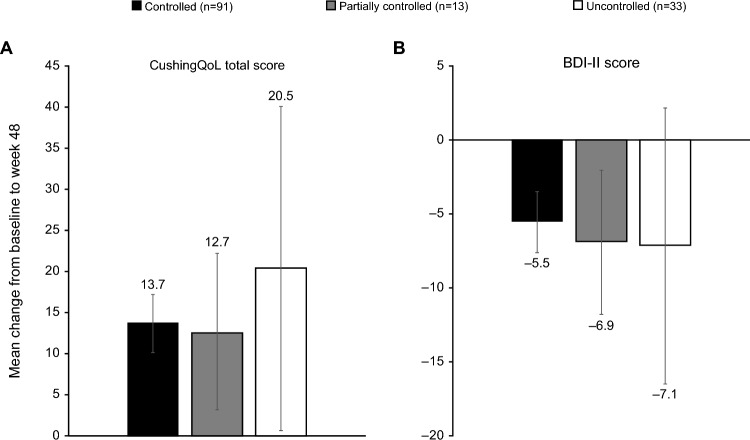
Mean (95% CI) change from baseline to week 48 in A) CushingQoL total score and B) BDI-II score by degree of mUFC control at week 48. CushingQoL scores range from 12 (worst) to 60 (best), and BDI-II scores range from 0 (best) to 63 (worst)

Clinically meaningful improvements in mean CushingQoL scores for the overall study population were reached at weeks 26, 30, 32, 34 and 48, and for mean change in BDI-II scores at weeks 24, 26, 28, 30 and 48 [12, 14, 15]. At week 24, numerical improvements in both mean (95% CI) CushingQoL and BDI-II scores occurred irrespective of whether patients had controlled mUFC (9.2 [6.1, 12.4] and − 3.6 [− 5.4, − 1.9], respectively), partially controlled mUFC (9.4 [0.9, 17.8] and − 6.8 [− 11.2, − 2.4]) or uncontrolled mUFC (10.1 [2.0, 18.2] and − 4.6 [− 11.2, 2.0]). A similar finding was observed at week 48, although the 95% CIs were notably wider for patients with uncontrolled mUFC (Fig. 5). There was a weak correlation between change in mUFC and change in CushingQoL score at week 24 (r = − 0.15, *P* = 0.0875), which was not evident at week 48 (r = 0.02, *P* = 0.8028). A weak correlation between change in mUFC and change in BDI-II score was observed at both week 24 and week 48 (r = 0.24,* P* = 0.0081 and r = 0.30, *P* = 0.0016, respectively).

## Discussion

Cushing’s syndrome, including Cushing’s disease, is associated with many clinical complications that affect multiple organ systems. The patients enrolled in LINC 3 typically presented with multiple underlying conditions and exhibited sometimes severe physical manifestations of hypercortisolism. Treatment with osilodrostat resulted in rapid and sustained normalisation of mUFC in many patients, with almost all patients achieving control of mUFC at least once during the study. Alongside controlling excess cortisol levels, medical therapy with osilodrostat resulted in improvements in multiple clinical features of hypercortisolism, which supports findings from trials of different medical therapies that act by reducing cortisol levels [16–23]. In the present study, to fully explore improvements in specific clinical signs and additional specific manifestations of hypercortisolism during osilodrostat therapy, a large number of clinical features were analysed over several time points. Initial improvements following osilodrostat treatment, as measured at week 24, were generally greater in patients with controlled or partially controlled mUFC than in patients with uncontrolled mUFC, which may be clinically important for some patients. It is important to note that the addition or adjustment of concomitant medications to treat comorbidities was permitted throughout the study and may have contributed to over- or underestimation of the observed improvements, such as in hypertension, dyslipidaemia and diabetes. The results of the present analysis also demonstrated that age, sex, race or time since diagnosis did not affect a patient achieving mUFC control during osilodrostat treatment.

Improving cardiometabolic parameters, such as blood pressure, weight, BMI and waist circumference, is an important aspect of care for patients with Cushing’s syndrome in order to reduce the risk of cardiovascular disease [24–27]. During osilodrostat treatment in patients with Cushing’s disease in the LINC 3 study, improvements in both SBP and DBP were observed, with over half of the patients with high blood pressure at baseline achieving normal SBP and DBP within 12 weeks, which remained within normal limits throughout the 48 weeks of the study. Furthermore, 40.0% of patients were able to stop or reduce their medication for hypertension during the study as prescribed at the discretion of the investigator. In addition, dose or number of antihypertensive medications was reduced or stopped in a numerically greater proportion of patients with controlled mUFC than in those with partially or uncontrolled mUFC, whereas a similar proportion of patients had an increase in dose or number of antihypertensive medications irrespective of mUFC control. Notably, improvements in SBP and DBP were numerically greater in patients with hypertension at baseline who did not receive antihypertensive medication during the study. Furthermore, patients who achieved control or partial control of mUFC at week 24 had numerically greater improvements in both SBP and DBP than those with uncontrolled mUFC. By week 48, when most patients had controlled mUFC (or could have achieved control of mUFC at some point during this time), it was not unexpected that improvements were evident irrespective of mUFC control. The correlation between change in mUFC and change in SBP or DBP from baseline was weak and indicated a weak relationship at week 48. Taken together, these findings highlight the benefits of controlling mUFC, which has the concomitant benefit of also improving blood pressure.

Although there was no correlation between change in mUFC and change in body weight, waist circumference or BMI, again, there was a tendency for patients with controlled mUFC at week 24 to have better outcomes. As weight and waist circumference are only gross indicators of fat mass, it is also possible that improvements occurred in visceral body fat that were not detected in this study. Overall, changes in cholesterol and triglycerides occurred irrespective of mUFC control, and there was no indication that improvements were related to the reduction in mUFC. Notably, some clinical features of hypercortisolism may take years to reverse, particularly when they have been present for some time [28].

Impaired glucose tolerance, including diabetes, is one of the main contributors to mortality identified in patients with Cushing’s syndrome [29–31]. During treatment with osilodrostat in the LINC 3 study, approximately 50% of patients with Cushing’s disease reduced their FPG levels to within normal limits, with some patients able to reduce or stop antidiabetic medications as prescribed at the discretion of the investigator; this was particularly notable in patients with controlled or partially controlled mUFC compared with those with uncontrolled mUFC. Improvements in FPG and HbA_1c_ were more evident in patients with controlled or partially controlled mUFC at both weeks 24 and 48. Furthermore, the correlation between change in mUFC and change in FPG from baseline was notable at both time points, indicating a possible direct relationship. Again, these data support the notion of aiming to control mUFC given the additional benefits to patients in relation to comorbidities.

Reduced BMD is common in patients with Cushing’s syndrome [2]. Even in patients with normal BMD, there is an increased risk of fractures as a result of cortisol excess [5, 32]. Data suggest that bone damage is reversible with control of hypercortisolism following surgery [2, 33]; however, data are limited on the effects of medical therapies [33]. Data from the 48-week core phase of the LINC 3 study indicated some improvement in BMD, which was more pronounced in male than female patients. It is possible that cortisol had not been at normal levels for long enough to have a clinically significant impact on BMD in all patients [28, 34]. Improvements in BMD may require longer-term medical treatment; patients with low BMD should be closely monitored and treated accordingly [5, 35].

Improvements in physical manifestations of hypercortisolism, including hirsutism in females, and HRQoL indicators (CushingQoL and BDI-II scores) generally occurred irrespective of the degree of mUFC control, suggesting that improvements may occur independently of the extent of cortisol reduction. This is similar to findings in prospective studies of pasireotide and levoketoconazole [16, 17]. For hirsutism in particular, the proportion of patients with improvements in rated severity score progressively increased, despite the potential for increases in testosterone levels following the initiation of osilodrostat [13]. Preliminary data suggest excess 11-oxygenated C19 steroids as the primary cause, the synthesis of which can be blocked by osilodrostat [36]. It is possible that a relationship could become more evident with longer follow-up [13, 37], considering the severity of hypercortisolism in patients at baseline in this study. Furthermore, as physical manifestations of hypercortisolism were assessed subjectively by different investigators, the findings should be considered with some caution. Interestingly, improvements in the rated score for proximal muscle atrophy at week 24 were numerically greater for patients with controlled or partially controlled mUFC than for those with uncontrolled mUFC. Data from the European registry have highlighted the prevalence of muscle weakness in patients with Cushing’s syndrome [29, 38], which was frequently reported in patients who died [29]. Improving muscle strength in these patients remains a challenge even in patients with surgical remission [39]; therefore, the early improvements in proximal muscle atrophy observed in patients with control of mUFC following osilodrostat treatment could be of clinical importance.

The current analysis is limited by the fact that patients were not evenly distributed between subgroups based on degree of mUFC control, as most patients achieved either complete or partial mUFC control from week 12 onwards [12]. As a result, some subgroups (eg partially controlled and uncontrolled mUFC) comprised fewer patients than different groups (eg controlled mUFC). Furthermore, it is important to acknowledge the limitations of measuring and guiding treatment decisions based on mUFC only, which was used to assess the primary endpoint in the current study. Measurements of mUFC can be subject to intra-patient variability [40] and can fluctuate among individuals according to their sensitivity to excess cortisol. As such, some comorbidities can persist despite improvements in hypercortisolism [2, 28]. It is probable that mUFC assessment alone is not sufficiently discriminative to fully reflect control of hypercortisolism, given the general improvements in clinical signs and additional manifestations of hypercortisolism irrespective of mUFC control by week 48, as well as the lack of correlation between mUFC and clinical outcomes. Further research into the relationship between clinical improvements and changes within the hypothalamic–pituitary–adrenal axis during medical therapy is warranted. Use of LNSC as a complementary measure for monitoring treatment response continues to be investigated [41], alongside changes in clinical outcomes, in patients who achieve control of both mUFC and LNSC.

In conclusion, improvements in clinical signs and additional specific manifestations of hypercortisolism associated with Cushing’s disease were observed during 48 weeks of therapy with osilodrostat, alongside the rapid and sustained control of mUFC. Normalisation of cortisol levels remains an important treatment goal for patients with Cushing’s disease, particularly when there are indications that controlled mUFC levels can be associated with the greatest clinical improvements.

## Supplementary Information

Below is the link to the electronic supplementary material.

Supplementary file1 (DOCX 24 KB)

## Data Availability

The datasets generated and analysed during the current study are not publicly available but are available from the corresponding author on reasonable request. Recordati Rare Diseases will share the complete de-identified patient dataset, study protocol, statistical analysis plan, and informed consent form upon request, effective immediately following publication, with no end date.
