# Supplementary material for: Improvement in clinical features of hypercortisolism during osilodrostat treatment: findings from the Phase III LINC 3 trial in Cushing's disease
Source: J Endocrinol Invest. 2024 May 2;47(10):2437–48. doi: 10.1007/s40618-024-02359-6 (PMC11392997; doi:10.1007/s40618-024-02359-6)
Supplement: Supplementary file 1 — Supplementary file1 (DOCX 24 KB) [file 40618_2024_2359_MOESM1_ESM.docx]

**Supplementary appendix**

**Improvement in clinical features of hypercortisolism during osilodrostat treatment: Findings from the Phase III LINC 3 trial in Cushing’s disease**

Rosario Pivonello *et al.*

**Supplementary Table 1. Overview of mUFC control by age, sex, race and time since diagnosis at 24 and 48 weeks**

| **Variable** | **n** | **Control, %  (95% CI)** | **Partial control, % (95% CI)** | **Uncontrolled, % (95% CI)** |
| --- | --- | --- | --- | --- |
| Week 24 |  |  |  |  |
| Age, years  ≤31  >31–<49  ≥49 | 36  63  38 | 63.9 (46.2, 79.2)  69.8 (57.0, 80.8)  68.4 (51.3, 82.5) | 19.4 (8.2, 36.0)  12.7 (5.6, 23.5)  13.2 (4.4, 28.1) | 16.7 (6.4, 32.8)  17.5 (9.1, 29.1)  18.4 (7.7, 34.3) |
| Sex  Male  Female | 31  106 | 77.4 (58.9, 90.4)  65.1 (55.2, 74.1) | 12.9 (3.6, 29.8)  15.1 (8.9, 23.4) | 9.7 (2.0, 25.8)  19.8 (12.7, 28.7) |
| Race  Caucasian  Asian  Black  Other | 89  39  4  5 | 71.9 (61.4, 80.9)  56.4 (39.6, 72.2)  75.0 (19.4, 99.4)  80.0 (28.4, 99.5) | 11.2 (5.5, 19.7)  23.1 (11.1, 39.3)  25.0 (0.6, 80.6)  0.0 | 16.9 (9.8, 26.3)  20.5 (9.3, 36.5)  0.0  20.0 (0.5, 71.6) |
| Time since diagnosis, months  ≤20  >20–<89  ≥89 | 36  67  34 | 55.6 (38.1, 72.1)  68.7 (56.2, 79.4)  79.4 (62.1, 91.3) | 13.9 (4.7, 29.5)  16.4 (8.5, 27.5)  11.8 (3.3, 27.5) | 30.6 (16.3, 48.1)  14.9 (7.4, 25.7)  8.8 (1.9, 23.7) |
| Week 48 |  |  |  |  |
| Age, years  ≤31  >31–<49  ≥49 | 36  63  38 | 58.3 (40.8, 74.5)  73.0 (60.3, 83.4)  63.2 (46.0, 78.2) | 13.9 (4.7, 29.5)  6.3 (1.8, 15.5)  10.5 (2.9, 24.8) | 27.8 (14.2, 45.2)  20.6 (11.5, 32.7)  26.3 (13.4, 43.1) |
| Sex  Male  Female | 31  106 | 80.6 (62.5, 92.5)  62.3 (52.3, 71.5) | 3.2 (0.1, 16.7)  11.3 (6.0, 18.9) | 16.1 (5.5, 33.7)  26.4 (18.3, 35.9) |
| Race  Caucasian  Asian  Black  Other | 89  39  4  5 | 68.5 (57.8, 78.0)  61.5 (44.6, 76.6)  75.0 (19.4, 99.4)  60.0 (14.7, 94.7) | 9.0 (4.0, 16.9)  12.8 (4.3, 27.4)  0.0  0.0 | 22.5 (14.3, 32.6)  25.6 (13.0, 42.1)  25.0 (0.6, 80.6)  40.0 (5.3, 85.3) |
| Time since diagnosis, months  ≤20  >20–<89  ≥89 | 36  67  34 | 55.6 (38.1, 72.1)  65.7 (53.1, 76.8)  79.4 (62.1, 91.3) | 2.8 (0.1, 14.5)  14.9 (7.4, 25.7)  5.9 (0.7, 19.7) | 41.7 (25.5, 59.2)  19.4 (10.8, 30.9)  14.7 (5.0, 31.1) |

Controlled, mUFC ≤ULN; partially controlled, >ULN but ≥50% reduction from baseline; uncontrolled, mUFC >ULN and <50% reduction from baseline, including patients who had discontinued or otherwise had missing mUFC values at a given visit. CI, confidence interval; mUFC, mean urinary free cortisol; ULN, upper limit of normal

**Supplementary Table 2. Correlations between change from baseline in mUFC and change from baseline in clinical parameters and HRQoL indicators at weeks 24 and 48**

|  | **mUFC week 24** | | **mUFC week 48** | |
| --- | --- | --- | --- | --- |
| **Parameter** | **r** | ***P* value** | **r** | ***P* value** |
| SBP, mmHg | –0.02 | 0.8326 | 0.20 | 0.0433 |
| DBP, mmHg | 0.02 | 0.8160 | 0.18 | 0.0715 |
| Weight, kg | –0.00 | 0.9991 | –0.04 | 0.6839 |
| BMI, kg/m^2^ | –0.02 | 0.7896 | –0.05 | 0.5755 |
| Waist circumference, cm | 0.09 | 0.3168 | 0.02 | 0.8285 |
| FPG, mg/dL | 0.25 | 0.0077 | 0.33 | 0.0009 |
| HbA_1c_, % | 0.23 | 0.0124 | 0.14 | 0.1612 |
| Total cholesterol, mmol/L | 0.08 | 0.3898 | 0.03 | 0.7850 |
| LDL cholesterol, mmol/L | 0.04 | 0.6572 | –0.06 | 0.5165 |
| HDL cholesterol, mmol/L | 0.08 | 0.3727 | 0.25 | 0.0104 |
| Triglycerides, mmol/L | 0.04 | 0.6975 | –0.04 | 0.6559 |
| CushingQoL total score | –0.15 | 0.0875 | 0.02 | 0.8028 |
| Standardised physical  problems score | 0.02 | 0.8485 | 0.06 | 0.5294 |
| Standardised psychosocial  issues score | –0.20 | 0.0248 | 0.01 | 0.9542 |
| BDI-II | 0.24 | 0.0081 | 0.30 | 0.0016 |

Highlighted boxes indicate *P*<0.05. BDI-II, Beck Depression Inventory II; BMI, body mass index; CushingQoL, Cushing’s Quality of Life Questionnaire; DBP, diastolic blood pressure; FPG, fasting blood glucose; HbA_1c_, glycated haemoglobin; HDL, high-density lipoprotein; HRQoL, health-related quality of life; LDL, low-density lipoprotein; QoL, quality of life; SBP, systolic blood pressure

**Supplementary Table 3. Mean percentage (95% CI) change from baseline to week 48 in BMD by degree of mUFC control at week 48**

| **Parameter** | **Controlled** | **Partially controlled** | **Uncontrolled** |
| --- | --- | --- | --- |
| L1–L4 lumbar spine – all patients | 2.9 (1.2, 4.6)  n=66 | 2.9 (0.6, 5.2)  n=11 | 5.3 (‒6.7, 17.4)  n=4 |
| L1–L4 lumbar spine – males | 3.9 (‒0.4, 8.2)  n=20 | 4.6 (NA)  n=1 | –  n=0 |
| L1–L4 lumbar spine – females | 2.5 (0.8, 4.1)  n=46 | 2.7 (0.2, 5.2)  n=10 | 5.3 (‒6.7, 17.4)  n=4 |
| Total hip – all patients | 0.5 (–0.9, 1.9)  n=66 | –0.2 (–3.0, 2.6)  n=10 | –1.0 (‒10.3, 8.3)  n=4 |
| Total hip – males | 2.1 (‒0.2, 4.3)  n=20 | –4.6 (NA)  n=1 | –  n=0 |
| Total hip – females | –0.1 (–1.9, 1.6)  n=46 | 0.3 (–2.6, 3.3)  n=9 | –1.0 (‒10.3, 8.3)  n=4 |

BMD, bone mineral density; NA, not assessable
